# Topical application of Chinese herbal medicine DAEP relieves the osteoarthritic knee pain in rats

**DOI:** 10.1186/s13020-019-0278-1

**Published:** 2019-12-09

**Authors:** Wing Sum Siu, Wai Ting Shum, Wen Cheng, Chun Wai Wong, Hoi Ting Shiu, Chun Hay Ko, Ping Chung Leung, Christopher Wai Kei Lam, Chun Kwok Wong

**Affiliations:** 1 Institute of Chinese Medicine, The Chinese University of Hong Kong, Shatin, NT Hong Kong SAR, China; 2 State Key Laboratory of Research on Bioactivities and Clinical Applications of Medicinal Plants, The Chinese University of Hong Kong, Shatin, NT Hong Kong SAR, China; 3 Faculty of Medicine and State Key Laboratory of Quality Research in Chinese Medicines, Macau University of Science and Technology, Macau, China; 4 Department of Chemical Pathology, The Chinese University of Hong Kong, Prince of Wales Hospital, Shatin, NT Hong Kong SAR, China; 5 Li Dak Sum Yip Yio Chin R & D Centre for Chinese Medicine, The Chinese University of Hong Kong, Hong Kong, China

**Keywords:** Chinese Medicine, Osteoarthritis, Pain, Topical treatment

## Abstract

**Background:**

The potential adverse effects of conventional oral pharmacotherapy of osteoarthritis (OA) restrict their long-term use. Topical application of a Chinese herbal paste for relieving OA knee pain can be effective and safe. However, evidence-based scientific research is insufficient to support its application worldwide. The aim of this study was to investigate the in vivo efficacy of a topical Chinese herbal paste on relieving OA knee pain and its underlying mechanism.

**Methods:**

An OA rat model was developed by anterior cruciate ligament transection (ACLT) followed by treadmill running. A herbal paste including Dipsaci Radix, Achyranthis Bidentatae Radix, Eucommiae Cortex and Psoraleae Fructus, named as DAEP, was applied topically on the knee joint of the rats (DAEP). The rats without DAEP treatment served as Control. Rats with surgery but without ACLT, treadmill running and DAEP treatment acted as Sham. The morphologic change of the knee joint was observed radiographically. Nociception from the knee of the rats was assessed using Incapacitent test and CatWalk gait system. The therapeutic mechanism was investigated by analyzing the gene and protein expression of inflammatory markers via qPCR and Western blot, respectively.

**Results:**

Radiographic images showed less destruction at the posterior tibial plateau of the DAEP group compared with the Control after 2 weeks of treatment. The static weight ratio and the gait parameters of the Control were reduced significantly via Incapacitance test and CatWalk gait analysis, respectively. DAEP treatment increased the Print Area and Maximum Intensity significantly compared with the Control. DAEP significantly suppressed the upregulation of gene expression of interleukin (IL)-6, tumor necrosis factor (TNF)-α, and inducible nitric oxide synthase (iNOS).

**Conclusions:**

DAEP exhibited its effect via the nuclear factor (NF)-κB pathway by suppressing the phosphorylation of IκB kinase αβ (p-IKKαβ) and cyclooxygenase-2 (COX-2) protein expression. This study provides scientific evidence to support the clinical application of the Chinese herbal paste on reliving OA pain.

## Background

Most conventional pharmacotherapy of osteoarthritis (OA) focus solely on symptomatic management. For instance, OA joint pain can be alleviated by paracetamol, oral or topical nonsteroidal anti-inflammatory drugs and weak opiates [1, 2]. Intra-articular glucocorticoid and hyaluronic acid have also been used to alleviate acute inflammation and knee pain in OA [3–5]. However, concerns regarding the potential adverse effects especially from long-term use, include gastrointestinal disturbances and cardiovascular risk [6–8]. Other symptom-soothing agents, such as glucosamine sulfate and chondroitin sulfate, may provide additional chondroprotection and impede OA progression [9–11]. Nevertheless, the efficacy of these supplements on ameliorating structural damages of OA joins remains controversial [12, 13].

Certain Traditional Chinese Medicine (TCM) formulae are generally believed to be effective against OA and are known to be non-toxic. These herbal formulae may shed lights on developing a novel agent for treating OA. We have studied the effect of an herbal formula on the proliferation of chondrocyte. This herbal formula was simplified from a classic prescription “Xu Duan Wan” from “Fu Shou Jing Fang”, which has been used for treating the soreness and weakness at waist and knee traditionally. Four herbs have been selected based on their popularity of use in traditional practice with the literature support for their bioactivities. They include Dipsaci Radix (Dipsacus asperoides C. Y. Cheng et T. M. Ai) (DR), Achyranthis Bidentatae Radix (*Achyranthis bidentata* Blume) (ABR), Eucommiae Cortex (*Eucommia ulmoides* Oliv.) (EC) and Psoraleae Fructus (*Psoralea corylifolia* L.) (PF). In this study, the four herbs were prepared as a herbal paste and named DAEP.

Numerous spontaneous and induced animal models have been developed to study disease onset and progression, as well as to test the novel therapeutic interventions of OA [14]. The induced animal models can be mainly divided by surgical manipulation or intra-articular chemical injection. Many surgically induced models have been reported and each of them relies on a combination of joint instability [15], altered joint mechanics [16] and inflammation to induce OA lesions. To evaluate the therapeutic potential of DAEP, we combined the first two factors and developed the rat osteoarthritis model by anterior cruciate ligament transection (ACLT) followed by treadmill running in order to confirm the successful development of OA at the knee joint of the rats.

The objectives of the current study were to evaluate the in vivo efficacy of the DAEP herbal paste on osteoarthritic condition and to obtain scientific data in support of its clinical application.

## Methods

### Herbal materials and authentication

Three batches of raw herbal materials of DR, ABR, EC and PF were purchased from a local TCM supplier in Hong Kong. Microscopic and morphological authentications were performed in accordance to the Chinese Pharmacopoeia [17] and Hong Kong Chinese Materia Medica Standards [18]. The presence of the standard chemical markers of each herb (DR: asperosaponin VI; ABR: beta-ecdysterone, ginsenoside Ro and chikusetsusaponin IV A; EC: pinoresinol diglucoside and PF: psoralen and isopsoralen) was authenticated using thin layer chromatography following the methods stated in the Chinese Pharmacopoeia. Authenticated voucher specimens of the herbal medicines were deposited in the museum of Institute of Chinese Medicine, CUHK, with voucher numbers: DR—3584; ABR—3581; EC—3583; PF—3582.

### Preparation of herbal paste

Herbal extracts were prepared by aqueous extraction, followed by ethanol extraction. Firstly, each herb (1 kg) was extracted by reflux for 1 h using 1 L distilled water. Following filtration, the filtrate was collected. Herbal residues were further extracted by reflux using 95% ethanol for 1 h and then filtered. The aqueous and ethanol extracts were combined and concentrated using a rotary evaporator until a viscous paste was form. A portion of each concentrated herbal extract was weighted before (wet weight) and after dried in an oven overnight (dry weight). The yield of extraction (total dry weight of each extract divided by 1 kg raw herb, multiplied by 100%) of DR, ABR, EC and PF was 46.7%, 53.1%, 11.6% and 24.4%, respectively. Considering that the topical administration of the DAEP herbal paste has not been studied before, it was prepared by mixing the four extracts at their simplest ratio 1:1:1:1 (in dry weight). Two percent of borneol (w/w) was added to increase the transdermal ability of the paste [19].

**Fig. 1:**
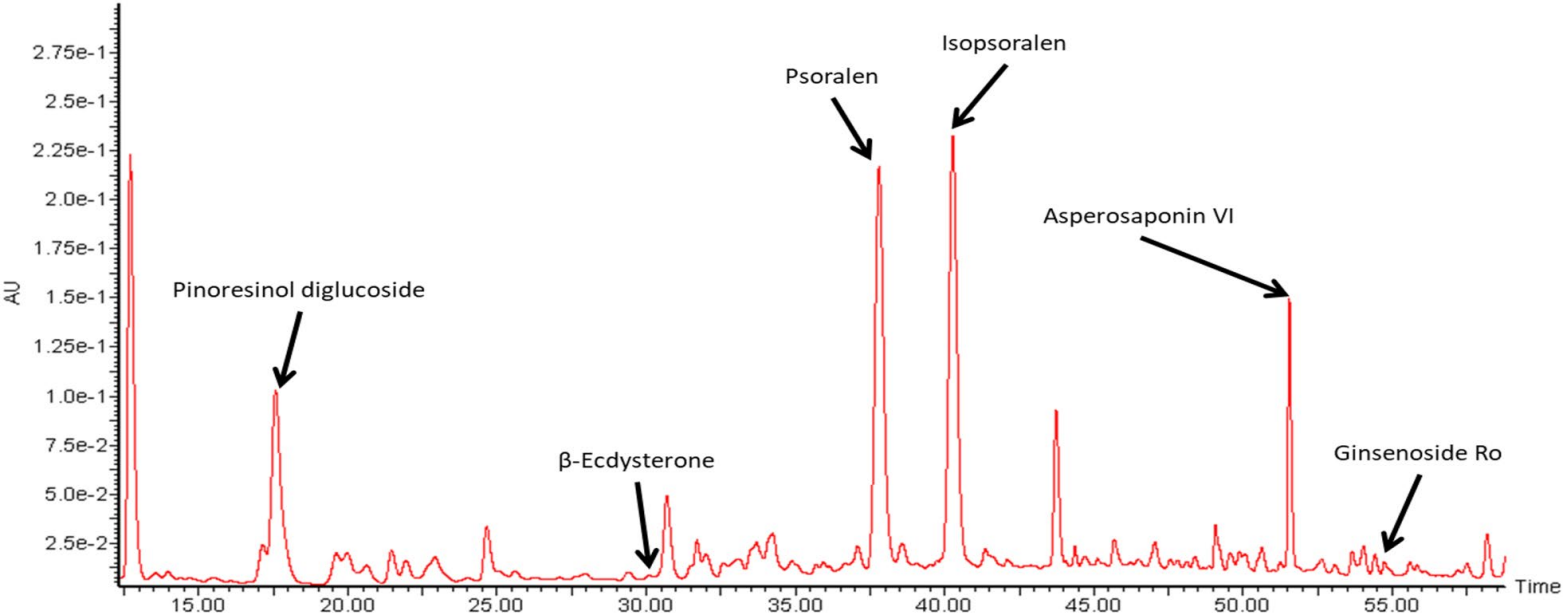
The UPLC profile of the DAEP herbal paste. Chemical profile of DAEP at 203 nm, mixed with 212 nm, 248 nm, 277 nm showing the peaks of all standard chemical markers, except chikusetsusaponin IV A

The abundance of each chemical marker for herbal authentication according to the Chinese Pharmacopoeia of the paste was determined quantitatively using ultra performance liquid chromatography (UPLC) (ACQUITY UPLC system, Waters Corporation, MA, USA; Table 1). The column used was Agilent ZORBAX Eclipse Plus C18 RRHD, 2.1 × 150 mm, 1.8 µm, accompanied with a guard column (Agilent ZORBAX Eclipse Plus C18 UHPLC Guard, 2.1 × 5 mm, 1.8 µm). The chromatographic separation was conducted at 40 °C under gradient conditions at a flow rate of 0.5 mL/min. The liquid chromatographic profile is as follows: Mobile phase: (A) 0.1% phosphoric acid in deionized water and (B) acetonitrile; Gradient: 0–5 min, 8% B; 5–11 min, 8–10% B; 11–17 min, 10% B; 17–32 min, 10–15% B; 32–41 min, 15–21% B; 41–48 min, 21–28% B; 48–58 min, 28–37% B. The column was flushed with 100% B for 3 min and re-equilibrium for another 3 min after each injection. UV 203 nm was used to determine chikusetsusaponin IV A and ginsenoside Ro (for ABR); UV 212 nm was used to determine asperosaponin VI (for DR); UV 248 nm was used to determine β-ecdysterone (for ABR), psoralen and isopsoralen (for PF); UV 277 nm was used to determine pinoresinol diglucoside (for EC). The concentration of each marker was calculated according to the standard curves of each individual chemical standard marker. The UPLC profile of the paste was shown in Fig. 1.

**Table 1:** Quantitative analysis on the chemical markers in the DAEP herbal paste and their transdermal property

Chemical marker	Herb	Conc. in paste (%)	Conc. in porcine skin (μg)	Conc. in receiving chamber (μg)	Molecular weight (g/mol)	Topological polar surface area (A^2^)
Ginsenoside Ro	ABR	0.15	6.64	0.00	957.12	312.0
Chikusetsusaponin IV A	ABR	0.01	12.39	0.00	927.09	292.0
Asperosaponin VI	DR	1.77	393.48	5.09	929.11	295.0
Pinoresinol diglucoside	EC	0.23	80.38	8.42	682.67	236.0
β-ecdysterone	ABR	0.02	4.82	0.51	480.64	138.0
Psoralen	PF	0.37	54.41	9.25	186.17	39.4
Isopsoralen	PF	0.33	45.11	9.51	186.17	39.4

### Transdermal property

Porcine ear skin was used as the membrane in a Franz chamber [20]. One gram of DAEP was loaded onto the skin which was mounted between the upper and the lower compartment (receiving chamber). After 24 h’ diffusion, the skin and the phosphate buffered saline (PBS) in the receiving chamber were collected. The skin was homogenized and then the chemical markers trapped inside were extracted with absolute methanol under sonication at 37 °C for 1 h and supernatant was then collected. The amounts of the chemical markers in the supernatant and the PBS were analyzed using UPLC as described above.

### Animal model

Animal experiments were approved by the Animal Experimentation Ethics Committee, The Chinese University of Hong Kong (Ref. no.: 16-259-MIS). Male Sprague–Dawley rats with mean weight 424 g were obtained from the Laboratory Animal Services Centre and maintained by the Laboratory Research Unit in the Prince of Wales Hospital, The Chinese University of Hong Kong. All rats were housed at a constant temperature of 21 °C with a 12-h light–dark cycle. Food and water were given ad libitum. The experimental procedure was started after 7 days of acclimatization.

The rats were anesthetized using an intramuscular (im) ketamine and xylazine cocktail, and pre-operative analgesia was administered with subcutaneous (sc) buprenorphine. The right knee joint cavity of the rat was exposed via a medial parapatellar incision. Anterior cruciate ligament transection (ACLT) was performed using micro spring-scissors and the medial meniscus was resected [15]. The incision on the skin was closed using suture. Post-operative analgesia with buprenorphine was given sc in the following 3 consecutive days every 12 h. All the animals were permitted to run uphill on a treadmill with inclination angle at 5° for 1 h every day after the surgical operation to ensure the development of knee OA [16].

The animals were randomly divided into three groups: DAEP group (with ACLT and meniscus resection, topical DAEP application; n = 12), Control group (with ACLT and meniscus resection, no topical DAEP application; n = 11) and Sham group (with same surgical procedure as DAEP and Control group but without ACLT and meniscus resection, no topical DAEP application; n = 5). DAEP herbal paste (0.5 mL) was applied topically around the knee in the DAEP group which started after the treadmill running on the day after ACLT. The paste was covered with a piece of gauze and secured by a thin plastic adhesive film to avoid licking by the rats. The application was renewed at 2-day intervals over the treatment period of 8 weeks.

### Assessments of OA

The progression of the OA was monitored radiographically. The level of pain of the animal in static and dynamic conditions was measured by the Incapacitance test and CatWalk gait analysis, respectively. All the assessments were performed on Day 0 (the day before ACLT, as baseline) and then biweekly. The animals were euthanized after 8 weeks of experiment and the articular cartilage from the knee joint was harvested for the analysis of gene and protein expressions using real-time quantitative polymerase chain reaction (qPCR) and Western blot, respectively.

### Radiographic assessment

Rats were anesthetized as mentioned above and then placed on the platform of the X-ray cabinet (UltraFocus^DXA^, Faxitron Bioptics, USA). Digital X-ray image from medial–lateral approach of the right knee was obtained biweekly.

### Incapacitance test

Rats were put into a holder specially designed to maintain it positioned comfortably on two separated sensor plates of an Incapacitance tester (Panlab Harvard Apparatus, USA). The static weight of each hindlimb that the rat applied on the two sensors was measured. Within the experimental period, the static weight of all rats increased together with their body weight. In order to exclude the interference of the body weight change, a static weight ratio (SWR) was calculated by dividing the static weight of the right hindlimb (OA limb) by the static weight of the left hindlimb (normal limb) of the same rat and multiplied by 100%.

### CatWalk gait analysis

Gait parameters of the freely moving rats were measured using the Catwalk gait analysis system (Noldus Information Technology, Wageningen, Netherlands). Briefly, rats were placed individually on the CatWalk glass platform and allowed to walk freely and traverse to and from one side to another side. The illuminated contact areas between the paws and the glass platform were recorded by a high-speed color video camera underneath the glass platform late. Each uninterrupted run with a minimum of 3-step sequence patterns were collected. Data of the right hindlimb from three compliant runs of each animal was analyzed biweekly over the 8-week treatment period. Based on the position, pressure, and surface area of each footprint, various gait parameters were quantified and analyzed through the CatWalk software 7.1. These parameters included: Stand Phase (time of paw contact with the glass plate in a step cycle); paw Print Area (surface area of complete print); paw Max. Intensity (in line with the degree of the maximum pressure a paw exerting on the glass plate); Swing Speed (computed by dividing the stride length by the swing phase duration) and Duty Cycle (the ratio between the stance duration and the complete step cycle duration).

### qPCR

The mRNA was extracted from the articular cartilage of proximal tibia using RNeasy Mini kit (Qiagen, Hilden, Germany). It was reverse‐transcribed into cDNA using Omniscript RT kit (Qiagen) with oligo‐dT primers (Life Technologies, CA, USA). For the qPCR, ABsolute QPCR Mix SYBR Green kit (Thermo Fisher) was used with a Light Cycler (Bio-Rad Laboratories Inc. CA, USA). The mRNA expression of interleukin (IL)-6, tumor necrosis factor (TNF)-α, inducible nitric oxide synthase (iNOS), cyclooxygenase-2 (COX-2) and matrix metalloproteinase 3 (MMP-3) was determined using the primer sequences listed in Table 2, with normalization to the housekeeping gene glyceraldehyde 3-phosphate dehydrogenase (GAPDH). The fold changes of gene expression were calculated using the 2^−ΔΔCt^ method.

**Table 2:** Rat primer sequences of target genes

Primer	Forward/Reverse	Sequence (5′ to 3′)
IL-6	Forward	ATCTGCCCTTCAGGAACAGC
	Reverse	AGCCTCCGACTTGTGAAGTG
TNF-α	Forward	CAGCCGATTTGCCATTTCATAC
	Reverse	GGCTCTGAGGAGTAGACGATAA
iNOS	Forward	CTCAGGCTTGGGTCTTGTTAG
	Reverse	TGTTGTTGGGCTGGGAATAG
COX-2	Forward	TCTCCAACCTCTCCTACTACAC
	Reverse	CTCCACCGATGACCTGATATTT
MMP-3	Forward	GGACCAGGGATTAATGGAGATG
	Reverse	CAGGGTCCAGAGAGTTAGATTTG
GAPDH	Forward	CAACGACCCCTTCATTGACC
	Reverse	CGCCAGTAGACTCCACGACAT

### Western blot

The articular cartilage harvested from the distal femur was lysed and homogenized. Protein samples (30 μg) were separated on 10% resolving SDS-PAGE gel electrophoretically and transferred to PVDF membrane (GE Healthcare, Buckinghamshire, UK). The membrane was blocked and then incubated with primary antibodies (Life Technologies). After washing, the membrane was incubated with secondary horseradish peroxidase conjugated antibodies (1:2000, Invitrogen, CA, USA). After the unconjugated secondary antibodies were removed, the signal was developed using chemiluminescence ECL assay Kit, and imaged on a Bio-Rad ChemiDoc™ XRS + imaging system (Bio-Rad). β-actin, a highly stable housekeeping protein and commonly used as an internal control in a variety of researches [21], was used to normalize the protein expression levels in the nuclear factor (NF)-κB pathway.

### Statistical methods

Data were expressed as mean ± standard deviation, unless otherwise specified. Comparisons among groups and time points were performed by Repeated Measure 2-way ANOVA in the Incapacitance test and the CatWalk gait analysis, or One-way ANOVA in the qPCR and Western blot assessments, both followed by Tukey’s multiple comparison test using GraphPad Prism 6. p < 0.05 was considered statistical significant.

## Results

### Abundance of chemical markers in the DAEP herbal paste

UPLC analysis showed that the concentration of asperosaponin VI from DR was the highest (1.77%) among all of the chemical markers in the DAEP paste, whereas chikusetsusaponin IV A from ABR was the lowest (0.01%) (Table 1). This concentration was too low to be distinguished clearly from the background noise of the UPLC profile of the paste when all the wavelengths of all chemical markers were mixed together (Fig. 1).

### Transdermal property of the DAEP herbal paste

UPLC analysis showed that asperosaponin VI and pinoresinol diglucoside from DR and EC, respectively are the most abundant markers trapped in the skin of the porcine ear (Table 1). However, the transdermal efficiency of psoralen, an isopsoralen from PF, was the highest, followed by β-ecdysterone and then pinoresinol diglucoside. Neither ginsenoside Ro nor chikusetsusaponin IV A from ABR penetrated the porcine ear skin and detected in the receiving chamber (Table 1). There was a negative correlation between the molecular weight/topological polar surface area and the permeability penetrating the skin of the markers (Table 1).

### Radiographic assessment on the development of the OA knee

**Fig. 2:**
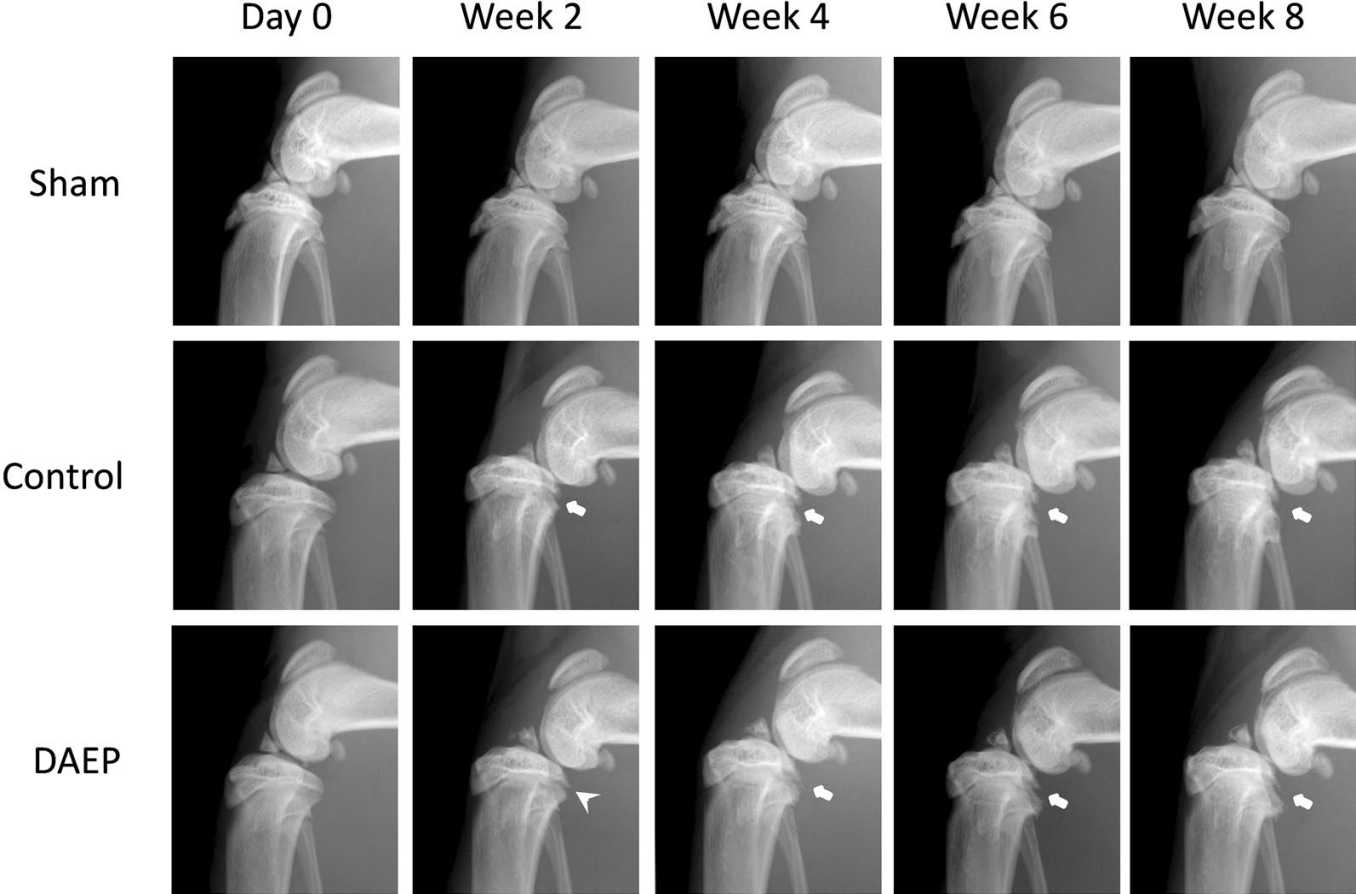
Radiographic images showing the development of OA at knee. Representative digital X-ray images from medial–lateral approach of the right knee were obtained before the ACLT (Day 0) and then biweekly thereafter (Week 2, 4, 6 and 8). Obvious destruction of the posterior tibial plateau is indicated by arrow. The destruction was reduced by the DAEP topical treatment at Week 2 as indicated by the arrowhead. Sham: the group of rats received surgical procedures to expose the knee joint cavity only, but without ACLT and meniscus resection, without treadmill running and DAEP treatment. Control: The group of rats received surgical procedures to expose the knee joint cavity, with ACLT and meniscus resection, with treadmill running but without DAEP topical treatment. DAEP: The group of rats received all the surgical procedures and treadmill running as Control, together with DAEP topical treatment

No adverse effect including body weight change was observed on the animal throughout the experiments. X-ray images showed that the femur of the Control group and DAEP group shifted backward (towards the posterior tibial plateau) after 2 weeks of ACLT and treadmill running (Fig. 2). A close contact between the femoral condyles and the posterior tibial plateau was observed. The posterior tibial plateau started destruction from Week 2 in the Control and DAEP group. However, the damage in the DAEP group was less than the Control group at this time point. The posterior tibial plateau of the Sham group remained intact throughout the experiment.

### DAEP prevented the reduction of static weight ratio against OA

There was no statistically significant change in the static weight ratio (SWR) in the Sham throughout the study (Table 3). A significant decrease in Week 2 (p < 0.0001) and Week 4 (p = 0.0013) occurred in the Control, whereas in Week 2 (p = 0.0066) only decreases in the DAEP were observed when compared with their own baseline (Week 0) value. When compared with the Sham, the SWR of the Control was smaller than the Sham at Week 4 and Week 8 (p = 0.0126 and 0.0271, respectively). No significant difference was found between the Sham and the DAEP at each time point.

**Table 3:** Static weight ratio measured by the Incapacitance test

Week	Mean (%) ± SD		
	Sham (n = 5)	Control (n = 11)	DAEP (n = 12)
0	92.30 ± 11.55	102.04 ± 9.24	100.60 ± 13.87
2	89.65 ± 15.78	74.38 ± 12.26***	83.80 ± 16.86**
4	102.52 ± 8.72	82.05 ± 11.53**^,#^	89.61 ± 12.09
6	100.75 ± 13.73	97.06 ± 12.19	89.93 ± 9.18
8	108.22 ± 11.73	89.71 ± 18.98^#^	93.29 ± 13.08

Sham: the group of rats received surgical procedures to expose the knee joint cavity only, but without ACLT and meniscus resection, without treadmill running and DAEP treatment. Control: The group of rats received surgical procedures to expose the knee joint cavity, with ACLT and meniscus resection, with treadmill running but without DAEP topical treatment. DAEP: The group of rats received all the surgical procedures and treadmill running as Control, together with DAEP topical treatment

** p < 0.01, *** p < 0.001 vs Week 0; ^#^p < 0.05 vs Sham

### DAEP improved the dynamic gait parameters of OA animal

**Fig. 3:**
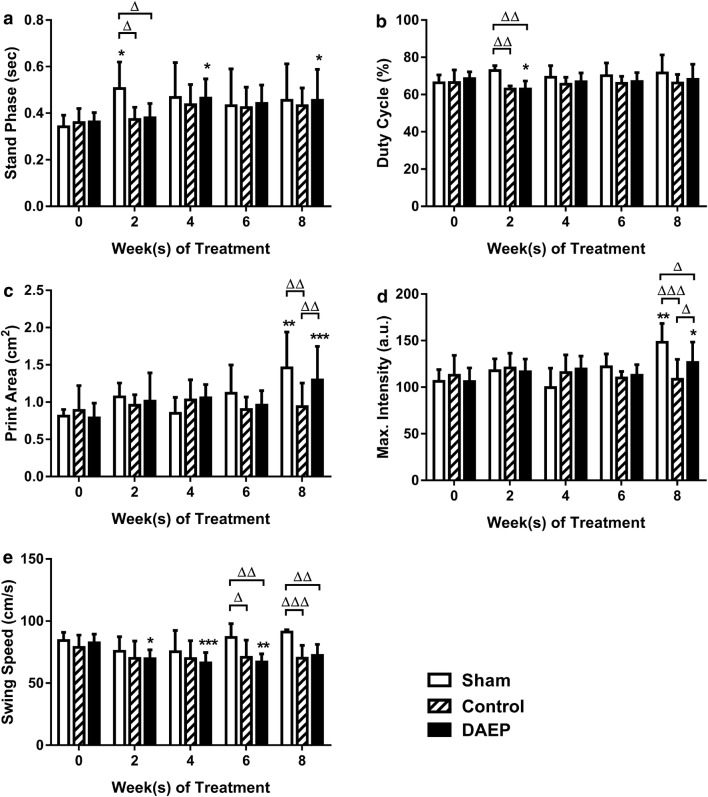
Comparisons of CatWalk parameters among groups throughout the experiment. Changes in gait parameters: **a** Stand Phase; **b** Duty Cycle; **c** Print Area; **d** Maximum Intensity; **e** Swing Speed. Results were shown in bar charts with mean + standard deviation; ^Δ^p < 0.05, ^ΔΔ^p < 0.01; ^ΔΔΔ^p < 0.001 (compared with the group denoted by n-zig-zag line); * p < 0.05, ** p < 0.01, *** p < 0.001 (compared with baseline (Week 0) of its own group). n = 5, 11 and 12 for Sham, Control and DAEP group, respectively

The Stand Phase and the Duty Cycle of both Control and DAEP groups were significantly lower than that of the Sham group after 2 weeks of OA induction. The Stand Phases of Control and DAEP groups were less than the Sham group by 0.133 s (26.24%, p = 0.0197) and 0.126 s (24.82%, p = 0.0268), respectively (Fig. 3a). The Duty Cycle of both Control and DAEP group was less than the Sham group by 9.91% (p = 0.0017 and p = 0.0014, respectively; Fig. 3b). At Week 8, the Print Area and the Maximum Intensity of the Control group were significantly lower than both Sham and DAEP group. The Print Area of the Control group was 0.521 cm^2^ (35.70%, p = 0.0028) and 0.360 cm^2^ (27.73%, p = 0.0088) smaller than the Sham and DAEP group, respectively. There was no significant difference between the Sham and the DAEP groups (Fig. 3c). The Print Area of the Sham and the DAEP groups was also significantly larger than their baseline value at this time point (p = 0.0031 for Sham, p = 0.0002 for DAEP). The Maximum Intensity of the Control group was lower than the Sham group by 26.72% (p < 0.0001) and the DAEP group by 14.08% (p = 0.0303), while the DAEP group was 14.70% lower than the Sham group (p = 0.0395; Fig. 3d). Similar to the Print Area, the Maximum Intensity of the Sham and the DAEP groups increased significantly compared with their baseline value (p = 0.0008 for Sham, p = 0.0158 for DAEP). Starting from Week 6, the Swing Speed of both Control and DAEP groups was significantly slower than the Sham group (Fig. 3e). The Swing Speed of the Control group was 15.95 cm/s (18.37%, p = 0.0149) and 21.16 cm/s (23.20%, p = 0.0008) slower than the Sham group at Week 6 and Week 8, respectively. Whereas in the DAEP group, the Swing Speed reduced by 19.93 cm/s (22.96%, p = 0.0014) and 18.96 cm/s (20.78%, p = 0.0025) compared with the Sham group at Week 6 and 8.

### DAEP suppressed the gene expression of inflammation markers in articular cartilage of OA knee

**Fig. 4:**
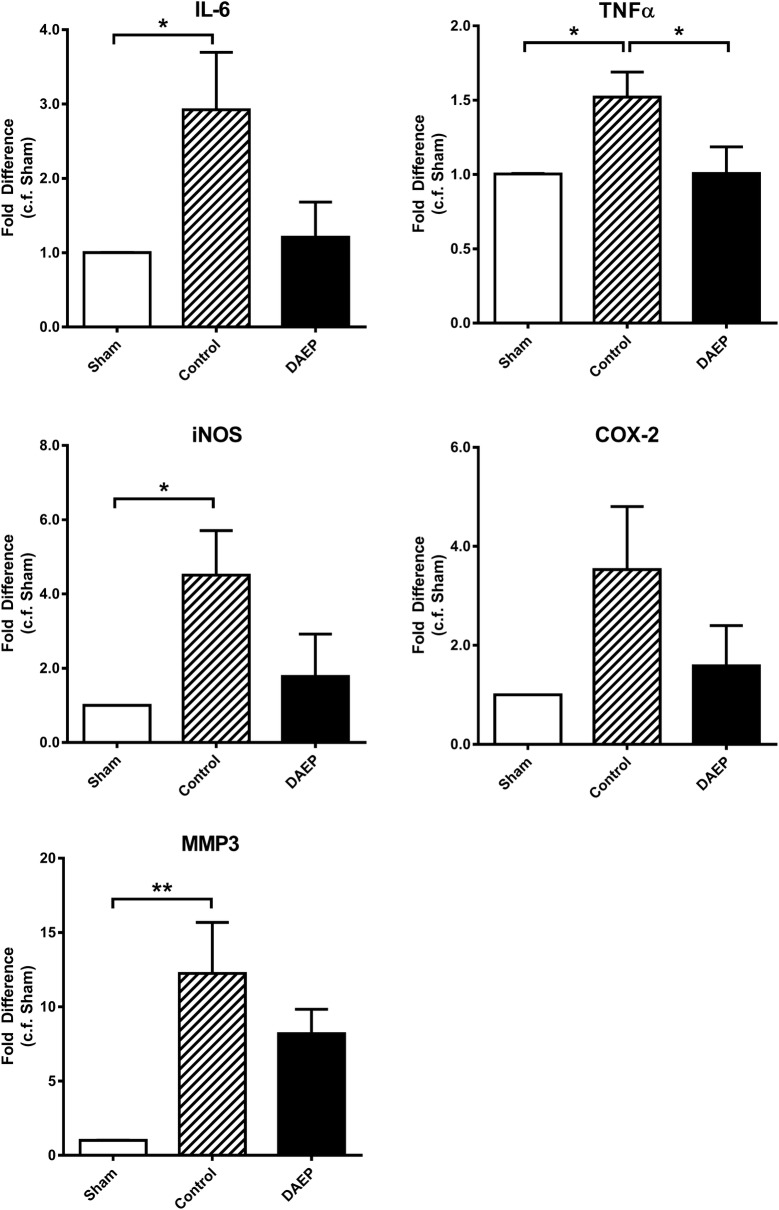
Effect of DAEP on gene expression of the articular cartilage of OA. Fold changes in IL-6, TNF-α, iNOS, COX-2 and MMP-3. Results are shown in bar charts with mean + standard error of mean (SEM); * p < 0.05, ** p < 0.01 (compared with the group denoted by n-zig-zag line). n = 5, 10 and 10 for Sham, Control and DAEP group, respectively

In the Control group, the mRNA expression of the inflammation markers IL-6, TNF-α and iNOS was upregulated significantly by 2.92 (p = 0.0389), 1.52 (p = 0.0405) and 4.50 (p = 0.0393) folds, respectively, compared with that of the Sham group (Fig. 4). COX-2 was 3.53-fold upregulated compared with Sham group (p = 0.1194). DAEP treatment significantly suppressed the upregulation of TNF-α during the OA development, with 1.51-fold less than that of the Control group (p = 0.0423). It also suppressed the upregulation of IL-6, with fold difference of 2.41 compared with the Control group, although not significant (p = 0.0710). There was no statistical difference in the mRNA expression of the four inflammation markers between the Sham and DAEP group (p = 0.9575, 0.9998, 0.8353 and 0.8828, for IL-6, TNF-α, iNOS and COX-2, respectively). Matrix degradation marker MMP-3 was significantly increased by 12.24-fold in the Control (p = 0.0034) but not in DAEP group (p = 0.0724) when compared with the Sham group (Fig. 4).

### DAEP suppressed the NF-κB pathway in articular cartilage of OA knee

**Fig. 5:**
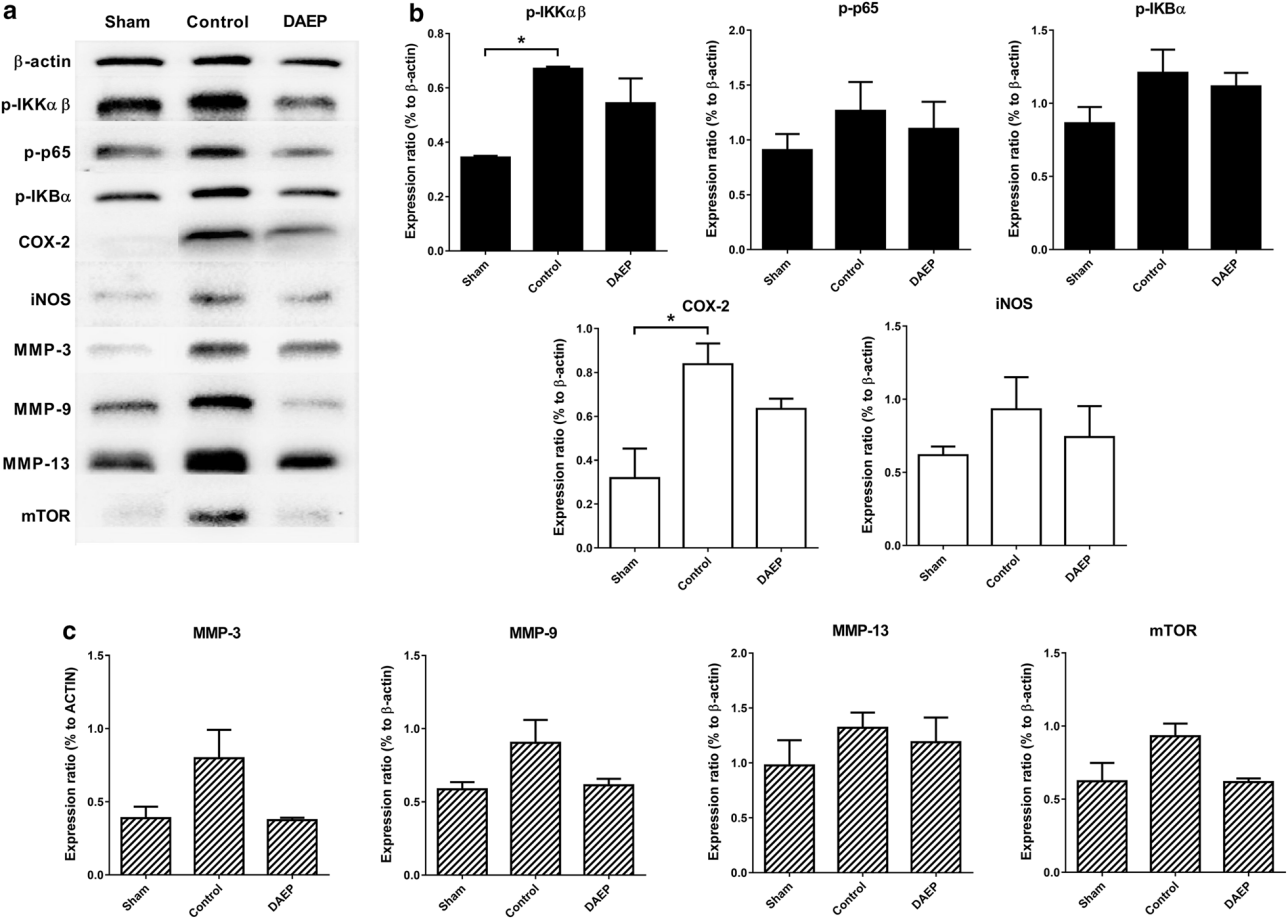
Effect of DAEP on protein expression in NF-κB pathway. Total protein of the articular cartilage from the distal femur of the OA limb was collected and then evaluated by Western blot. β-actin was used as an internal control (**a**). Protein expression relating to inflammation (**b**) and matrix degradation (**c**) was quantified by densitometry using ImageJ software and normalized to the β-actin level. Results are shown in bar charts with mean + standard error of mean (SEM), * p < 0.05 compared with the Sham. n = 3, 6 and 6 for Sham, Control and DAEP group, respectively

The protein expressions in the NF-κB pathway of the Control group were increased after the rats experienced ACLT and treadmill running (Fig. 5a). p-IKKαβ was upregulated significantly by 95.22% (p = 0.0115). p-p65 and p-IkBα were also upregulated, although without statistical significance (p = 0.5383 and 0.2007, respectively). The NF-κB pathway downstream protein iNOS and COX-2 were also upregulated and the later was increased by 2.63-fold significantly (p = 0.0231) (Fig. 5b). Proteins for degradation of extra-cellular matrix including MMP-3, MMP-9 and MMP-13 as well as mammalian target of rapamycin (mTOR) for the autophagy of chondrocyte, were also upregulated in the Control group (Fig. 5c) compared with the Sham group (p = 0.1189, 0.1338, 0.4729 and 0.1102, respectively). The expression of all these proteins was reduced in the DAEP group when compared with the Control group (p = 0.1075, 0.1708, 0.8896 and 0.1037, respectively).

## Discussion

Successful development of OA knee rat model by combining ACLT followed by treadmill running could be confirmed from radiographic assessment, with obvious deterioration at the posterior tibial plateau of the rat observed at 2 weeks post-operation. With the topical DAEP treatment, the deterioration was reduced compared with the Control.

The Incapacitance test (static weight bearing test) in the current study represents an unsurpassed method for assessing spontaneous pain in the hind limbs. Although the static weight quantified reflects the spontaneous pain of the rat due to the OA knee, the change in the static weight in a longitudinal study can be affected by the change of the body weight of the animal. As long as the animal grows within the experimental period, the static weight increases. Therefore, self-comparison of the static weight of the affected limb alone throughout the experiment could not reflect the degree of OA knee pain precisely. A ratio of the static weight of right hind limb (OA limb) to that of the left hind limb (normal limb), viz. SWR, is therefore better to illustrate the degree of the spontaneous pain in a longitudinal study. It could minimize the interfering factor of the change of the body weight of the animal. In this study, the significant decrease in the SWR of both Control and DAEP at Week 2 post-operation demonstrated that the rats in these two groups were affected by pain from the right knee which is in concordance to previous publication [22]. The authors reported that the percentage weight on the ACLT hind limb of the rats declined significantly at the first several weeks and then remained stable from Week 5 post-operation. On the other hand, at Week 4 post-operation, the SWR of the Control, but not the DAEP group, was significantly lower than the baseline value. This observation illustrated that the rats in the Control group still bore their body weight by their left leg dominantly at this time point, while the ones in the DAEP started to bear their body weight by both legs. In addition, significant difference in SWR between the Sham and the Control group was found at Week 4 and 8 post-operation. This result also elucidated that the rats experienced ACLT and treadmill running but without DAEP treatment produced prolonged weight-bearing asymmetry. The Incapacitance test showed that topical treatment of DAEP relieved the knee pain of the rats when they were at a resting posture (Table 3).

The nociception from the OA knee of the rat at motion was reflected by the Catwalk gait analysis. The Stand Phase and the Duty Cycle of the Control and DAEP group were shorter dramatically when compared with the Sham group 2 weeks post operation. These parameters indicated that the rats were unwilling to touch the glass platform of the Catwalk by their OA limb during walking at the first 2 weeks. The Swing Speed of the Control and DAEP group was slower than that of the Sham group significantly at Week 6 and Week 8, demonstrating that the rats in these two groups tried to minimize the contact frequency on the glass platform by the OA limb. Corresponding to the result of the Incapacitance test, the Print Area and the Maximum Intensity of the Control group were significantly smaller than that of the Sham group at Week 8. These findings showed that the rats in the Control group avoided supporting their body weight by their OA leg during walking. Other studies also showed that the percentage of total ipsilateral paw print intensity of rats received intra-articular injection of collagenase in the knee was lower than the control group [23]; and the area/pressure of the ipsilateral paw of OA animals induced by monosodium iodoacetate (MIA) injection were significantly different from the control group [24]. Importantly, both the Print Area and the Maximum Intensity of the DAEP group were significantly higher than those of the Control group at Week 8, illustrating that DAEP topical treatment ameliorated the OA knee pain of the rats at walking at this time point (Fig. 3).

The pain relieving effect of the DAEP paste could be contributed by the anti-inflammatory effect of the ingredients. Although OA has been regarded as a non-inflammatory arthritis, inflammatory processes do play a significant role in the progression of the articular cartilage damage in OA patients [25, 26]. Patients with OA knee joint suffer from more severe pain during normal activities and under resting conditions if they have a higher synovitis score [27]. Aberrant elevation of pro-inflammatory nitric oxide (NO) involves in the nociception and pain can contribute to the functional disability of OA [28]. Inhibitors of NO synthesis may have analgesic effects for treating inflammatory and neuropathic pain [29], and reduce nociception. Moreover, OA-related inflammatory cytokines TNF-α and IL-6 are present in the joints of the experimental OA animal model [30, 31]. As shown in Fig. 4, topical DAEP treatment significantly suppressed the upregulated local gene expression of IL-6, TNF-α, iNOS, and COX-2 in the OA knee. It indicated that DAEP reduced the production of NO and pro-inflammatory cytokines, leading to the analgesic effect. The results from the Western blot further illustrated that the anti-inflammatory effect of DAEP on OA was through suppressing the intracellular NF-κB pathway which has an impact on nociceptive transmission and processing. The classical (canonical) NF-κB pathway with the activation of IKKα/IKKβ/IKKγ-NEMO heterodimeric complex can result in phosphorylation and subsequent degradation of IκB molecules via the ubiquitin–proteasome system [32]. Our results demonstrated that DAEP treatment could downregulate the gene expression of TNF-α, with the suppression of the upstream p-IKKαβ expression, leading to the inhibition of the downstream COX-2 expression in OA condition.

Among all the chemical markers in the DAEP paste, asperosaponin VI from DR was the highest in concentration. Asperosaponin VI is an anti-inflammatory agent with anti-inflammatory and anti-nociceptive activity via the downregulation of NO generation [33]. Our studies on the effect of herbal paste containing DR on fracture healing demonstrated that the paste significantly reduced NO production [34]. Asperosaponin VI can also inhibit the expression TNF-α, IL-1β and COX-2, Akt and IκB kinase phosphorylation and NF-κB activation [35]. In this study, the skin penetration ability of asperosaponin VI was not the highest among the other chemical markers, which is similar to previous report [36]. However, the addition of borneol in the DAEP paste can enhance the skin penetration ability of asperosaponin VI because it can increase the cellular uptake and synergize the pharmaceutical effect of drug [36]. Therefore, asperosaponin VI from DR might show its anti-inflammatory effect on the OA knee in this study even though its transdermal efficiency is not the highest. Actually, a herbal formula containing DR for topical application is effective to relieve the pain from paw edema on rat [37].

β-ecdysterone from ABR is also an anti-inflammatory agent. By inhibiting the NF-κB signaling pathway, it suppressed the NO production by attenuating the iNOS protein expression [38], and IL-1β-induced apoptosis and inflammation [39]. Our clinical trial also demonstrated that a topical herbal paste containing ABR relieved the pain and improved the foot and ankle function of patients suffering from plantar fasciitis effectively [40]. Pinoresinol diglucoside from EC, which was a highly permeable chemical marker in the DAEP herbal paste, upregulated the expression of heat shock factor 1 and heat shock proteins to protect cells against stress conditions such as inflammation and oxidative stress [41].

The most permeable chemical markers in the DAEP herbal paste were psoralen and isopsoralen from PF. In a recent OA study, psoralen significantly inhibited TNF-α- induced MMPs and inflammatory cytokine production from synoviocytes, as well as activated cartilaginous extracellular matrix synthesis in vitro [42]. Consequently, it protected and activated chondrocytes and therefore attenuated MIA-induced OA in a rat model [42]. In an in vitro study, psoralen significantly suppressed T helper type 2 (Th2) cytokines such as IL-4, IL-5 and IL-13 and therefore regarded as a critical component of PF for its in vivo therapeutic effects on airway hyperresponsiveness and inflammation of asthma [43]. Similarly, isopsoralen downregulated TNF-α and IL-6 expression levels of lipopolysaccharide (LPS)-activated murine macrophage and in the bronchoalveolar lavage fluid of the acute lung injury mice induced by LPS, via the inhibition of the NF-κB and mitogen-activated protein kinase (MAPK) pathways [44]. Isopsoralen also showed significant inhibition effect on NO release [45].

## Conclusions

The present study confirmed the in vivo efficacy of the topical application of DAEP herbal paste on relieving OA knee pain because of its anti-inflammatory ingredients within the paste targeting the suppression of the NF-κB pathway. Moreover, DAEP reduced the MMPs and mTOR expression during OA development, thereby implying that DAEP may retard the OA progression. Since no adverse effects have been observed on the animal throughout the study, this topical herbal formula should be safe to be used. Together, this study provided strong scientific evidence for future clinical trial using this herbal formula for the topical treatment of OA.

## Data Availability

The datasets used and/or analyzed during the current study are available from the corresponding author on reasonable request.

## Abbreviations

OA: osteoarthritis
TCM: Traditional Chinese Medicine
DR: Dipsaci Radix
ABR: Achyranthis Bidentatae Radix
EC: Eucommiae Cortex
PF: Psoraleae Fructus
DAEP: the name of the herbal paste/the group of rat treated with the herbal paste
ACLT: anterior cruciate ligament transection
UPLC: ultra performance liquid chromatography
PBS: phosphate buffered saline
SWR: static weight ratio
IL-6: interleukin-6
TNF-α: tumor necrosis factor alpha
COX-2: cyclooxygenase-2
MMP: matrix metalloproteinase
NF-κB: nuclear factor-κB
IκB: inhibitory protein of kappa B
IKK: IκB kinase
iNOS: inducible nitric oxide synthase
mTOR: mammalian target of rapamycin
